# The Architecture of an Automatic eHealth Platform With Mobile Client for Cerebrovascular Disease Detection

**DOI:** 10.2196/mhealth.2550

**Published:** 2013-08-09

**Authors:** Xingce Wang, Rongfang Bie, Yunchuan Sun, Zhongke Wu, Mingquan Zhou, Rongfei Cao, Lizhi Xie, Dong Zhang

**Affiliations:** ^1^College of Information Science and TechnologyBeijing Normal UniversityBeijingChina; ^2^School of Economics and Business AdministrationBeijing Normal UniversityBeijingChina; ^3^MD. Department of neurosurgeryBeijing Tiantan HospitalCapital Medical UniversityBeijingChina

**Keywords:** cerebrovascular, eHealth platform, Ball B-Spline, statistical segmentation, volume rendering

## Abstract

**Background:**

In recent years, cerebrovascular disease has been the leading cause of death and adult disability in the world. This study describes an efficient approach to detect cerebrovascular disease.

**Objective:**

In order to improve cerebrovascular treatment, prevention, and care, an automatic cerebrovascular disease detection eHealth platform is designed and studied.

**Methods:**

We designed an automatic eHealth platform for cerebrovascular disease detection with a four-level architecture: object control layer, data transmission layer, service supporting layer, and application service layer. The platform has eight main functions: cerebrovascular database management, preprocessing of cerebral image data, image viewing and adjustment model, image cropping compression and measurement, cerebrovascular segmentation, 3-dimensional cerebrovascular reconstruction, cerebrovascular rendering, cerebrovascular virtual endoscope, and automatic detection. Several key technologies were employed for the implementation of the platform. The anisotropic diffusion model was used to reduce the noise. Statistics segmentation with Gaussian-Markov random field model (G-MRF) and Stochastic Estimation Maximization (SEM) parameter estimation method were used to realize the cerebrovascular segmentation. Ball B-Spline curve was proposed to model the cerebral blood vessels. Compute unified device architecture (CUDA) based on ray-casting volume rendering presented by curvature enhancement and boundary enhancement were used to realize the volume rendering model. We implemented the platform with a network client and mobile phone client to fit different users.

**Results:**

The implemented platform is running on a common personal computer. Experiments on 32 patients’ brain computed tomography data or brain magnetic resonance imaging data stored in the system verified the feasibility and validity of each model we proposed. The platform is partly used in the cranial nerve surgery of the First Hospital Affiliated to the General Hospital of People's Liberation Army and radiology of Beijing Navy General Hospital. At the same time it also gets some applications in medical imaging specialty teaching of Tianjin Medical University. The application results have also been validated by our neurosurgeon and radiologist.

**Conclusions:**

The platform appears beneficial in diagnosis of the cerebrovascular disease. The long-term benefits and additional applications of this technology warrant further study. The research built a diagnosis and treatment platform of the human tissue with complex geometry and topology such as brain vessel based on the Internet of things.

## Introduction

### Background

Cerebrovascular disease is the number one threat to people’s health, especially for people over the age of 50. Statistics indicate that as many as 15 million people die from cerebrovascular disease every year worldwide [1]. Cerebrovascular disease is characterized with long latency, quick onset, intense demand of timely rescue, and long follow-up treatment time. China is a big county with a large population and vast geographical areas. If the government depends solely on the traditional center hospital mode to diagnose and prevent the cerebrovascular diseases, it would result in heavy burdens on hospitals and increase difficulty on patients to see doctors. Development of medical imaging technologies, ultrasound, computed tomography angiography (CTA), magnetic resonance angiography (MRA), and 3-dimensional (3D) rotational angiography (3DRA) have achieved higher precision and lower cost on cerebrovascular disease imaging. By using the general medical image, we can extract the cerebrovascular area. This technology is easily accepted by people because it is non-invasive, efficient, and has no side effects. Brain scans have become part of the routine physical examination for employees in China every year. As a result, every worker has accumulated a lot of image data from computed tomography (CT) or magnetic resonance imaging (MRI) scans. Our platform is constructed with efficient use of these data, which can achieve cerebrovascular automatic query, segmentation, reconstruction, testing, and evaluation. The platform can combine information technology and cerebral medical technology to promote the development of eHealth technology and create the new situation of the brain medical care in China.

### The Internet of Things and Platform Design

Professor Kevin Ashton proposed the concept of the Internet of things (IOT) first. The basic idea of this concept is the pervasive presence around us of a variety of things or objects, such as radio frequency identification devices (RFID), tags, sensors, actuators, mobile phones, etc. Through unique addressing schemes, they are able to interact with each other and cooperate with their neighbors to reach common goals [2]. RFID technology and sensor technology are applied to everyday objects to form “the Internet of things". Facing the problem of cerebrovascular disease, the application of the Internet of things in the medical field can realize the monitoring of severe patients, daily health care of ordinary users, detailed analysis of the popularity of cerebrovascular disease, and in depth teaching about cerebrovascular disease in the medical school. Traditional cerebrovascular detection and analysis of brain tissue image sequences rely on the CT or MRI machine. Doctors observe the instrument screen image or film by eye to determine cerebral vascular disease with high subjectivity and poor spatiality. In addition, cerebral blood vessels make up a low proportion (<5%) of the brain tissue. Using the manual method to extract the region of interest has some unexpected results, such as incomplete area, unreachable viewpoint, fatty tissue scattered points affecting the observations and so on. At the same time, the observers must rely on graphics workstations connected with the CT or MRI.

In 2005, the conference of Asia Pacific Electronic Health held in China launched domestic research in applications of the Internet of things in medicine. In 2006, the national 863 special project carried out telecommunication network, television network and computer network (3NET) fusion of electronic health information systems research. In 2009, the research group conducted a preliminary trial in ChangNing, Shanghai for the electronic case and health care combining with the phone, television, and Internet [3]. Now, the eHealth platform of IOT is mainly used in health care (eg, alcohol dependence [4] and personal health record [5]), disease recovery (eg, cardiac rehabilitation [6,7]), and social public domain (eg, impact disease detection [8], psychological health intervention [9], cancer detection [10], and the vicious epidemic monitoring [11]). Before the research on the Internet of things was applied in the medical field, some electronic application platforms of vascular diseases had existed, but most of them were facing virtual operation and virtual endoscope with a single computer, which worked mainly for the services of the central hospital. Correlation studies on vascular virtual operations such as the CathSimsystem [12], CathI heart operation system [13], and the NeuroCathsy system Singapore [14] mainly focused on model construction and force feedback calculation in virtual operation. Since then, other important works have been done linking vascular diseases with IOT, such as monitoring the heart disease [15] and the retinal vessel detection [16]. These platforms currently do not completely use all the available data. The development history of domestic cerebrovascular health platform of cerebral vessels indicate that platforms are mostly used in the primary application areas of electronic health for the processing and remote transmission of electronic case, doctors’ offsite health information, and the popularization of health knowledge, but it is less used in the human abnormal detection and the development of chronic disease. The current domestic cerebrovascular health platform is limited to use by technically trained staff in the hospital. Our platform is designed such that anyone can use it. Our platform combined computer technology with the medical imaging technology to detect and analyze cerebral vessels. It can extend the virtual display and medical assistance advisory services to the model analysis and quantitative calculation, achieving wider application through the Internet media.

This paper is organized as follows. In the Methods section, we illustrate the level architecture of the platform in our work and describe the 8 main functions of the platform in detail. The client part of the platform will also be illustrated in this section. We then present our results on clinical datasets and give the analyses.

## Methods

### System Platform Design

**Figure 1:**
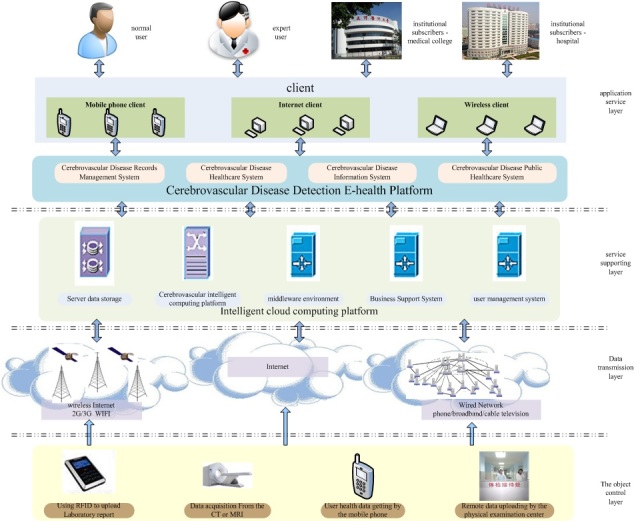
The hierarchy of the Internet of things eHealth platform about cerebrovascular treatment.

The architecture of what has been accepted as the Internet of things still follows the traditional network architecture. It is divided into three levels [17]: (1) generalization perception network, (2) communication infrastructure of integration network, and (3) the supporting system of pervasive application service. These are usually simply referred to as the perception layer, network layer, and application layer. The eHealth platform in this paper adopts the 4 layer-platform architecture proposed by Professor Ma Huadong [18](Figure 1): (1) object control layer, (2) data transmission layer, (3) service supporting layer, and (4) application service layer.

Based on this framework, we realized the extended application facing the cerebral vascular medicine. The object control layer achieves the physical object perception and data acquisition, including various tests identified by the RFID, sensor nodes of widespread deployment, the wireless sensor network, a remote digital imaging and communications in medicine (DICOM) data transmission, and a variety of test data provided by the natural human and examination center. Data transmission layer provides the transparent information transmission through various cable networks and wireless networks. Service supporting layer mainly provides the intelligent processing of the data acquisition from the network and service-applying platform, including intelligent server management and cloud computing platform. Application service layer provides service by transmitting the information to the content. The server based on the cloud platform is the core part of the system, which completes the basic functions of the system. The client offers the doctor user interface and the common user interface. Brain image data of the common user obtained from center hospital and medical center is transmitted to medical server platform through the network. The server finishes the data storage and reconstruction of cerebral vascular. It can also make the reconstruction results and automatic detection results feedback to the common user through the network. The doctor browses the patient information through the login interface, which can also provide diagnostic information and fuse the system diagnosis result to send back to the common user. The user can use the mobile phone to query brain image data to finish the automatic detection of cerebral vascular health, and receive the diagnosis and treatment recommendations given by the doctor to realize remote independent medical service. Unlike the traditional remote diagnosis system, the main evaluation means and diagnosis method of this system are relying on the system itself instead of the direct judgment of the doctor. The whole platform is an automatic system. The brain vessel can be segmented, reconstructed by the platform automatically, and the suspicious region of cerebrovascular disease can also automatically be detected by the system. Because the doctor only needs to provide the corresponding aid support, this greatly reduces the workload of doctors and develops the application degree of the system.

**Figure 2:**
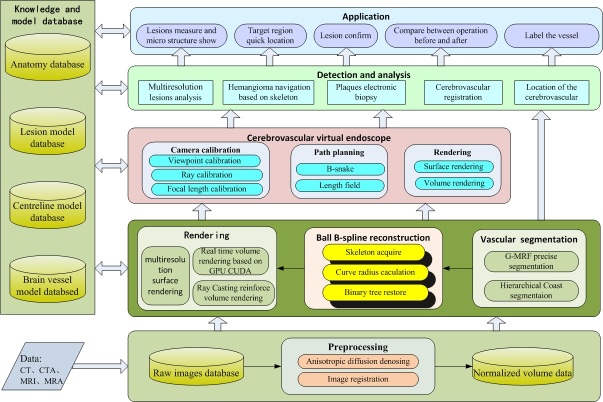
The function structure of medical service platform about cerebrovascular disease.

It can be found that the main work associated with cerebrovascular disease is realized at the application service layer. The system function structure is shown in Figure 2. The overall frame can be summarized as 4 layers and 6 databases. The implementation scheme includes 5 steps. The 4 layers are the data support layer, the basic technology layer, the service technology layer, and the application system layer. The six databases are the original image database, the normalized volume database, the vascular lesions model database, the center line of blood vessels model database, the brain vessel model database, and the physiological anatomy knowledge database.

The main flow of the 5 steps is as follows:

1. Construction of image database. CT or MRI cerebral images containing basic information of the patient are obtained from the hospital and medical center. After denoising and normalization, the raw data can be used to construct the normalized body database to facilitate subsequent processing.
2. Brain vascular segmentation model. The segmentation of the point cloud model, the geometric model of brain vessel data, and segment brain vessel tissue are realized through the statistical method.
3. Construction of cerebrovascular model. The cerebrovascular skeleton is extracted based on the cerebrovascular point cloud data, and calculate radius of cerebral vessel. The Ball B-spline is used to reconstruct the brain vessel. At last, the brain vessel model and reconstruction model are imported into the database to provide the prior knowledge for the segmentation.
4. Rendering of cerebrovascular model. Graphics processing unit (GPU) compute unified device architecture (CUDA) allows for real-time and accelerated rendering, while fuse surface rendering and volume rendering finishes the mixed rendering of hierarchical model.
5. Cerebrovascular virtual wandering and lesions automatic detection. The model database and prior knowledge database are combined to realize the automatic tagging of organs. The suspicious lesions can be confirmed as below based on the physical parameters of organs according to the organization intensity, shape information, and prior knowledge to carry out the electronic biopsy. Auxiliary detection and analysis of lesions is designed with volume of interest (VOI) enhance analysis, realize focal and quantitative analysis through a variety of means. The rapid positioning and the navigation of target area can be determined by the prior knowledge automatically, and the corresponding path is established to facilitate intra operative real-time navigation. The comparison between the preoperative and postoperative is evaluation of the focal change before and after surgery through the registration between the prior model and the segmentation or reconstruction results.

This system adopts the Client/Server (C/S) structure based on ASP.NET platform, which uses the AJAX technology, SQL Server 2008 database management system, and ADO.NET database access technology. The front-end JS framework and corresponding components adopt Ext and the server is coded using Visual Studio 2005.

### The Main Functions of Cerebrovascular Disease Detection eHealth Platform

The server adopts ASP.NET to provide WEB services through building the .NET platform and uses ADO.NET to realize the persistence of access layer. It can obtain more precise OOP features to use the .NET platform, such as inheritance, encapsulation, and reflection. At the same time, .NET provides many powerful functions components, improves the speed of development and enhances the stability and correctness of the system. In addition, the .NET platform can improve the manageability, availability, safety, and other aspects of the overall system. In addition, the powerful visual studio is used to develop the system in order to obtain a more efficient development process.

### Whole Introduction of Server System Functions

The system at the server is used for the segmentation and reconstruction of cerebral CT and MRI image, which mainly adopts the statistical algorithm to finish cerebral vascular segmentation and the method of B-spline surface for the reconstruction of vessels. Methods of volume rendering and surface rendering are used to display the results after segmentation and reconstruction, and the virtual endoscope is used to achieve automatic detection of cerebral vascular diseases. In addition, the platform can realize the transformation between DICOM, raw, and BMP data. The overall characteristics of the system are: (1) processing many kinds of common data structure (raw, DICOM, BMP etc), (2) using a variety of segmentation methods to make the segmentation results accurate and effective, (3) powerful ability of user interaction, (4) friendly interface, convenient operation, (5) choosing a variety of display strategies and comparing the visual effects, (6) strong practicability and the results of every step can be viewed in real-time.

### Cerebrovascular Database Management

**Figure 3:**
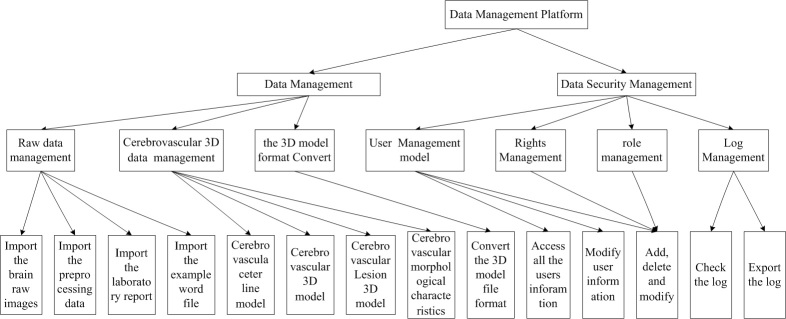
Platform of database management.

**Figure 4:**
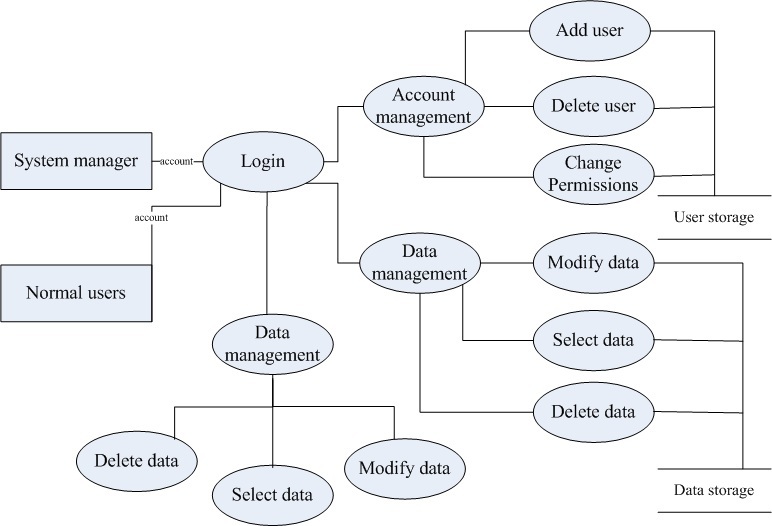
Data flow diagram of database management.

MRI and CT imaging equipments are used to realize the data acquisition and the data of this part is mainly obtained from picture archiving and communication system(PACS) and examination center of hospital. In Figure 3, the visual database management module is divided into two subsystems, the data management and database security management. Combined with a relational database management system, all kinds of data involved in the study are organized and managed. This module contains three major functions: function of database link, function of data management, and function of database security management. The data involved in this project mainly includes: the 2-dimensional (2D) original medical image data, the 2D medical image data after preprocessing, data of cerebral vascular segmentation model, data of cerebral vascular skeleton, data of cerebral vascular remodeling model (Ball B-spline data) as well as basic attribute information data of the basic personal. Database link function mainly completes the link and disconnection operation of front interface and the backstage database. The login password management is involved in implementation. The function of database security management includes: add, delete, and modify the role; user information and user permissions; modification of personal information; information view of all users; and journal management. At the same time, cerebral vessel data management system based on relational database and the generalized system of safety certification are established. Data flow chart of data management system is shown in Figure 4.

### Preprocessing of Brain Image Data

**Figure 5:**
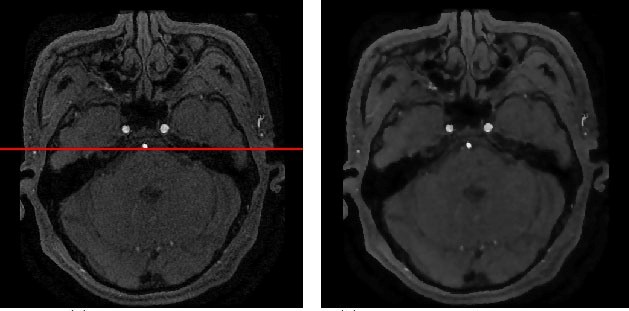
Level noise reduction. Left: Original image; right: Anisotropic diffusion result.

**Figure 6:**
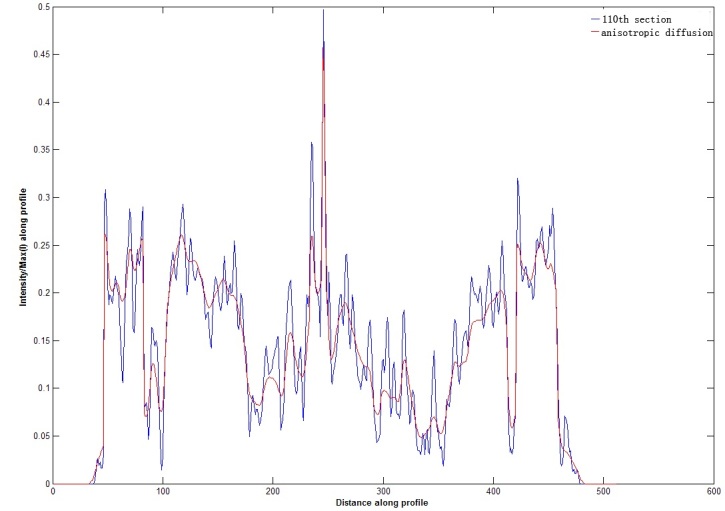
Intensity contrast of images before and after anisotropic diffusion.

Since brain images are affected by acquisition equipment, acquisition environment, sports of acquisition object, and the transmission process, a lot of noise will be produced, which directly affects the image quality and is extremely easy to cause the error analysis and error segmentation results of blood vessels. Therefore, the denoising, enhancement, and standardization of the cerebral image are the foundation for further analysis and processing. There is a lot of Gauss noise and random noise in cerebral images, so filtering image to reduce the interference of noise is a very common strategy, which is mainly divided into spatial filtering and frequency domain filtering. A hierarchical noise reduction processing method is used to achieve progressive process of 3D image sequences. In the processing of low order noise reduction, the template method is mainly used, where the directional weighted median (DWM) filter to remove the random impulse noise, and the Gauss template to remove other noise. In the processing of higher order noise reduction, the multi-scale method and the anisotropic diffusion method [19] is used, which can better maintain the vessel edge while removing noise. At the same time, Laplace sharpening is used in the images to highlight the edge of brain image. Robert operator is used to extract the edge of the scalp in the brain image, because the brain skeleton is fixed and the epidermis only has slight deformation, which can be regarded as rigid body. The method based on the edge feature can realize the brain image registration and interpolation normalization. The accuracy of subsequent processing would be improved. Figure 5 is the result after second order noise reduction on the 110^th^ picture of example data. After first order Gaussian filter and DWM filter and second order anisotropic diffusion filter, the difference between two images is not obvious. The red lines are the 248th line of 110^th^ image of example data. Figure 6 is the comparison chart of pixel intensity distribution in the same line position (red) between the original image and the corresponding picture after anisotropic diffusion filter. The intensity distribution of original image is shown with blue curve, and the intensity distribution after the anisotropic diffusion is shown with red curve, where x-axis represents the distance between the current pixel and left starting point on red line, y-axis represents the intensity value after intensity value of current pixel mapped (divided by 928) to [0,1] according to the intensity maximum. It can be seen in large gradient area of blue curve (the upper and lower steep slope section of curve, namely the picture boundary location), red curve appropriately retained the big wave crest and big wave trough of the blue curve. In the area shown by the blue curve where the intensity is flat, red curve showed obvious smooth processing. This shows that the hierarchical noise reduction processing method remove the noise and at the same time keeps the detailed information of edge.

### Image Viewing and Adjustment of Window Width and Window Level

CT and MRI image can display the distribution of some physical quantity in space. CT shows the distribution of intensity information, while MRI obtains the electromagnetic signal from human body. The human tissue CT values range from −1000 to +1000, which is a total of 2000 degrees, but the resolution of the MRI is lower than CT. The human eye can only distinguish 16 intensity levels, so if all the degrees are shown, only a small part can be recognized by human eye, which will lose a lot of details. In order to improve the details of the organizational structure displayed and distinguish two tissues whose difference is small, we need to adjust the contrast and the display range of the image. Therefore, cerebral vessels will be better displayed through the adjustment of window width and window level. The window width is the display range of the CT or MRI image. The organizational structure in this range is divided into 16 degrees (intensity) from white to black according to the values of physical quantity acquired. Window level refers to the average value of window width in the CT or MRI image. Because CT (MRI) values of different tissues are different, the best choice to observe the fine structure of the CT or MRI image is to choose the CT (MRI) value of the organization as the center to scan, and this center is set to the window level. So the contrast is strong and resolution intensity is close to the organization or structure. When we see the image through the default window width and window level, the image often looks fuzzy, but we can see the cerebral vessels clearer by adjusting the window width and window level.

### Image Cropping, Compression, and Measurement

Either CT data or MRI data of cerebral image is usually large images. A lot of useless data of cerebral image occupy more memory of the system, which is time consuming. In order to get better region of interest (ROI) presentation, we can appropriately crop and compress images to improve the processing speed while ensuring the effect. At the same time, we need to measure the image in order to obtain some parameters of the image. Image processing functions of visualization toolkit (VTK) are used to achieve image cropping, compression, and measuring. Display of 3D data requires the establishment of space coordinate system. When data are cut, since the target result is still a cube, we choose the two peaks of body diagonal, which can be easily used to calculate other peaks of the cube. The image cropping is completed by excluding the points out of the cube. Image compression first requires that the 3D compression ratio be set artificially and to filter the points according to the proportion, such as setting the ratio 4 at the axial direction, and then at this direction we can choose one in each 4 points (up, down, left, and right). Image measurement obtains the coordinates, length, angle, and other basic information by using the measurement tools provided by VTK. The appropriate data cutting and compression can enhance the image and speed up the image processing.

### Brain Vascular Segmentation

The cerebral vascular image acquired is often about the whole brain, but only the vessel part should be used to realize the feature extraction and target recognition. Thus, the segmentation of cerebral vessel is the basis for the further work. The system platform needs network transmission to guarantee the amount of calculation and . The existing cerebrovascular automatic detection eHealth platform was designed mainly for the patients, and the interaction of setting the right initial seed points and defining contour model and the curve evolution equation requires more medical knowledge, so it obviously does not suit the requirements. Instead, the statistical model method without manually inputting the parameters and the initial position is suitable for our platform [20,21].

**Figure 7:**
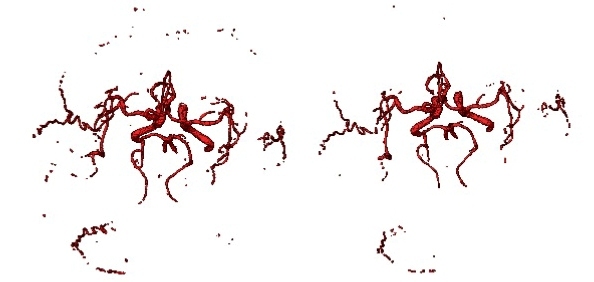
Comparison of G - MRF and double Gaussian model segmentation result. Left: G - MRF segmentation result; right: double Gaussian model segmentation result.

**Figure 8:**
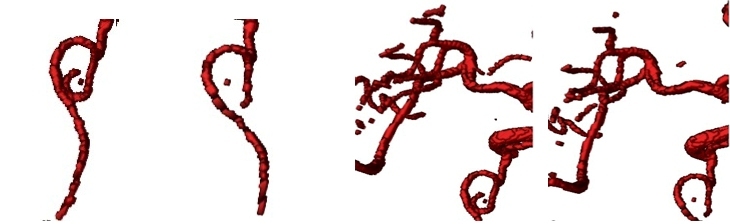
Comparison of segmentation details between G-MRF model and double Gaussian model of example data. From left to right: Detail 1 of G-MRF segmentation; detail 1 of Double Gauss segmentation; Detail 2 of Double Gauss segmentation; Detail 2 of G-MRF segmentation.

An automatic statistical approach based on the Gaussian-Markov Random Field Model (G-MRF) is employed. The voxels are classified as either blood vessels or background noise by a finite mixture of two Gaussian distributions. 3D MRF is employed to improve precision and those parameters are estimated by the Stochastic Estimation Maximization (SEM) algorithm, which converges to the true likelihood under a large lattice. The MRF field embeds the spatial neighborhood information to the parameters statistical model and increases regional morphology information in the gray statistic information. With this method, more three-level brain vessels can be achieved. The cerebral vascular segmentation algorithm based on SEM hybrid model solves the traditional slow convergence and local minimum problem of expectation maximum (EM) algorithm. From Figures 7 and 8, compared with double Gauss model [22], our algorithm can effectively segment the main branch and the surrounding smaller branches of brain vessel, and its convergence speed improves greatly than the traditional EM algorithm.

### 3D Cerebrovascular Model Based on Ball B-Spline Curve

Ball B-Spline Curves (BBSC) [23] are skeletons based on the parametric solid model, which are particularly suitable for representation of tubular shapes. They can be viewed as the extensions of B-Spline Curves from which many properties are inherited [24].

**Figure 9:**
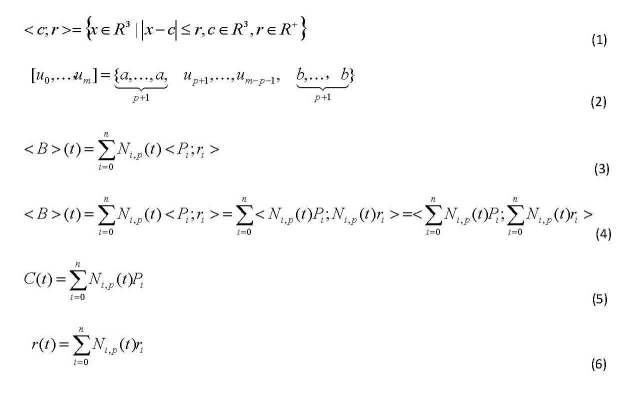
The equations of the paper.

A ball is defined in equation (1) in Figure 9. Here *c* is the center of the ball and *r* is the radius. *N*_*i,p*_ *(t)* is the i-th B-Spline basis of degree *p* with knot vector equation (2) in Figure 9. The ball B-Spline Curve is defined in equation (3), Figure 9, where *P*_*i*_ is called control point, *r*_*i*_ is called control radius. The number of control points, the dimensionality of the knot vector and the degree *p* are not independent, they satisfy equation (4) (Figure 9), so a Ball B-Spline Curve can be viewed as two parts, the center curve (or skeleton; equation (5), Figure 9), a 3D B-Spline curve, and the radius function equation (6) in Figure 9, a B-Spline scalar function. Owing to the perfect symmetry property of balls, the curve *C(t)* constructed from the centers of balls is exactly the skeleton of the 3D region represented by the BBSC.

**Figure 10:**
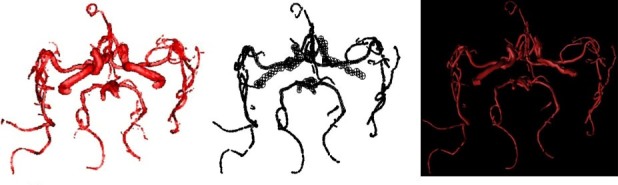
Reconstruction of segmentation result. From left to right: Model of Cerebrovascular segmentation; The corresponding radius of cerebral vessels; The cerebral vessels model represented by ball B-spline.

Ball B-Spline modeling methods are very similar to those of B-Spline. Interpolation, approximation, and deformation, which are typical modeling methods in B-Spline, can be easily extended to BBSC by applying algorithms of B-Spline curve and function to the center curve and the radius function of BBSC respectively. When a series of points {*Q*_*i*_}, *i*=0,…,*m* on the central curve and their corresponding radius {*R*_*i*_} are given, a BBSC whose center curve passes these data points {*Q*_*i*_} and whose maximum radius is {*R*_*i*_} can be obtained by interpolation. In order to obtain BBSC interpolation, we used the B-Spline interpolation method to interpolate the center curve, as well as B-Spline scalar function method to interpolate the radius respectively. Other methods can be implemented similarly. For more properties and algorithms about BBSC, please refer to [23,24]. The dataset size of cerebrovascular model based on Ball B-Spline Curve is far smaller than triangle mesh, point cloud, and other representations when representing a 3D tubular object. Typically, the size of medical image data of one patient is tens or hundreds of megabytes. The BBSC model, which only costs several kilobytes space for a patient’s data, is an excellent way to store and transmit data in telemedicine, and suitable for our eHealth platform. The reconstruction results are shown in Figure 10.

**Figure 11:**
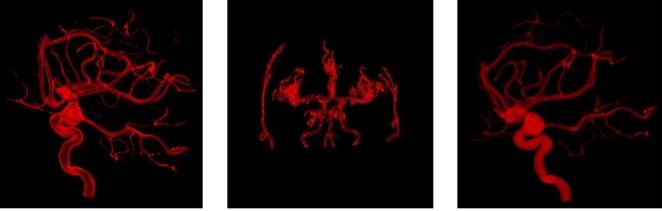
Volume rendering results of cerebral vessels. From left to right: The contour enhancement based on gradient; The contour enhancement based on curvature; The boundary enhancement based on depth.

**Figure 12:**
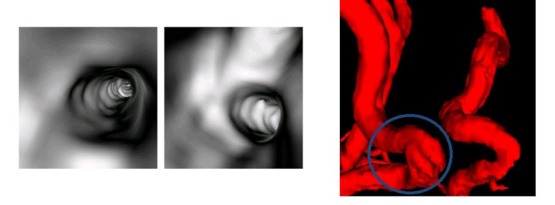
The virtual endoscope and automatic detection result. From left to right: Cerebrovascular virtual endoscopic results of the lumen;Cerebrovascular virtual endoscopic results of vessel cross region; Automatic detection results of cerebral vessels.

### Cerebrovascular Rendering Technology

The cerebral vascular image data is based on the 3D scalar field, the visualization method can be divided into surface rendering and volume rendering algorithms. Marching cube algorithm is a typical surface rendering algorithm, which uses the patch rules to approximate surface and extracts the surface information from the object of interest. Compared with the surface rendering algorithm, the volume rendering algorithm will regard all the information of 3D data field as input, which can vividly display the internal structure of rendering objects and have obvious advantages in data mining of intrinsic hidden information. The expression of such small cerebral vessels of complex structure is more effective than the surface rendering. But cerebral vascular structure can be restored with great detail using this technology, which can show its spatial adjacency details and have important clinical value in the diagnosis of cerebrovascular diseases. In the process of volume rendering, some ideographic means of the feature enhancement are proposed to characterize the complex spatial structure and continuous topological relationship of the brain vessel. Based on the high quality ray casting volume rendering with CUDA, we propose the silhouette enhancement based on the curvature to perform the silhouette width information, the boundary enhancement based on the depth to make the near boundary clearer than the distant boundary. The technology of depth cue based on stereoscopic is displayed by using the gradually changed color and stereoscopic display, which provides intuitive understanding for the observers and provides support for research and analysis of disease [25]. The characteristics of ray casting algorithm is beneficial to preserve detail and render a high quality image. It is especially suitable for the 3D imaging of the rendering region with fuzzy characteristics and voxel features of high correlation, which meets the need of presentation of cerebral vessels. The curvature can reflect the degree of local concave and convex surfaces of the objects, which represents the structure information. In order to enrich the contour information, we bring in the width factor based on the idea of the contour enhancement of curvature. So we can effectively increase the opacity of contour area to highlight contour and increase the interactivity and flexibility of contour enhancement through the adjustment of the opacity of vascular data. The junction of different substances will be highlighted in the volume rendering of cerebral vessels, which makes the physical distribution clearer in the result image. In order to make the rendering result containing depth information, a higher edge enhancement can be implemented in the part of smaller depth, while a lower edge enhancement can be implemented in the part of larger depth, which makes the near boundary clearer than the distant boundary.

### Cerebrovascular Virtual Endoscope and Automatic Detection

In the key technology of virtual endoscope navigation, path planning is the base of virtual endoscope mirror to realize navigation. Because manual navigation is very difficult, time-consuming, and easy to get lost, the path needs to be carefully planned and the viewpoint must match the planned path. This project studied the interactive central path planning algorithm based on B-Snake model, and the algorithm does not require prior segmentation of the organs to directly extract the center path of tubular organ in the original image. The algorithm makes full use of the advantages of the active contour model and B-spline function and omits the internal energy of the traditional Snake model in the B-Snake model, which reduces the parameters of the model and is easy to control. The mobile polyhedron center method is used in the algorithm. The mobile polyhedron is defined in the chamber of the vessel, and the external force of the B-snake is defined as the force stretching the polyhedron centroid to move towards the center of the vessel chamber, which is robust to the noise of image. The curve extracted by this model is smooth and continuous, which meets the requirements of virtual endoscope navigation. At the same time, local support and continuity of the B-spline curve are used to obtain the smooth central path with less time. The vascular diameter information can be obtained after the vessel reconstruction using the Ball B-spline. The vessel radius is measured according to different sequence at the same vessel. If the difference between vessel radius and the adjacent node radius exceeds the threshold, it is thought area of lesion and needs to be marked. Based on the identification, it can help doctors and patients to judge.

### Introduction of the Client

The system adopts C/S model to achieve the overall function. In order to ensure information security and reduce the amount of communication, the fat server/thin client model is used. The main functions of the system are concentrated in the server, and the client provides remote uploading and browsing of data, disease detection, and other functions.

### Introduction of the Mobile Client Realization

Mobile cerebral vascular eHealth client mainly provides remote uploading of data, data browsing, segmentation, reconstruction, and automatic detection functions. The mobile client on android platform is realized by the JS and can be secondly developed based on remote desktop protocol (RDP). Because the preview and operate of the graphic and image are necessary in our system, the RDP and virtual network computing (VNC) protocols are more suitable for these requirements. Comparing these two types of protocols, we can see that RDP has the advantages of less transmission flow than VNC, fast response, low operation delay, and is more applicable to the Windows system. Our system uses the basic RDP protocol and implements a second phase of development and optimization to better adapt to the mobile client. In the mobile client, the display and manipulate of the cerebral vascular system is necessary, so the RDP can be cut and optimized for this use. The transmission rate of mobile client can be chosen from 8, 16, 32 bit color. The sound support, printer support, communication port support, and clipboard support are removed to improve transmission rate. The file system is transformed to support the definition, which is convenient for the directional transmission of vascular images. At the same time, the cerebral vascular image on the client is chosen to transmit to the specified directory of the server, and the task is divided to build different folders to store files. The vascular initial data is analyzed by the operating procedures. The mobile client displays the result in 3D, and allows users to interact with the client and set the parameters to upload them to the server through the mobile phone client.

### Introduction of the Network Client

Network client provides the functions of uploading, data browsing, cutting, segmentation, reconstruction, and automatic detection of remote brain image slice data. Network client is mainly applied in the implementation of HTML and XML, because the transmission and display of 3D model is implemented at the client through the Virtools software. The server uses the Visual Studio, the client uses VC to create Windows Sockets support and increases CRequest Socket to realize the communication between client and server. The message response function OnReceive is added in the CRequest Socket class for receiving the data sent by the server. Network client implements the client-side application based on the browser and allows the entire system only deploy and update in the center of the WEB server, which eliminates the necessity that any part of the application is explicitly deployed to the client computer. At the same time, this model enables users to efficiently and conveniently make the application public to a large scale of various external audiences, which realizes the online inquiries, large-scale information publication and service sharing function.

## Results

So far, our system has stored the brain CT data or brain MRI data of 32 patients, and the relevant system has been preliminarily tested and applied in cranial nerve surgery of First Hospital Affiliated to the General Hospital of People's Liberation Army and radiology of Beijing Navy General Hospital. Our system also has some applications in medical imaging specialty teaching of Tianjin Medical University. We use SQL Server 2008 to construct cerebrovascular database and the submission system of remote data management at the server. The application software platform at the server background is Microsoft ASP.NET, Visual Studio 2005, and VTK 5. The system is in the process of testing. The hardware system is Inter (R) Xeon (R) E5410 2.33GHz, graphics card is NVIDIA GeForce GTX 570, the GPU architecture is CUDA 3.2. A group of DICOM medical data is used to demonstrate the clinical effect of the system, including 136 time of flight (TOF) MRI image, which we call example data. The maximum interval is 2.1mm and the minimum is 0.7mm between images. The diameter of reconstruction is 200mm. Other data related to privacy of patients are not provided here.

**Figure 13:**
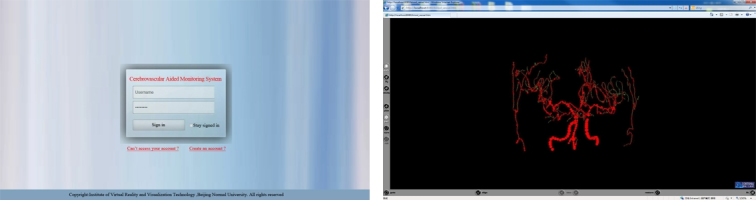
The interface of the network client .Left: Landing interface at remote Client; right: The Ball B-spline structure at remote client.

**Figure 14:**
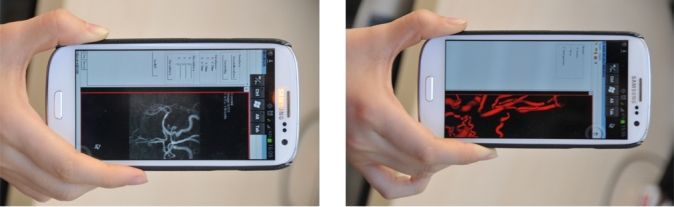
The interface of the mobile phone client. Left: MIP projection data displayed at mobile phone Client; right: Segmentation results at mobile phone Client.

The function screenshots of each part of the system are as follows: Web-based remote submission system interface is shown in Figure 13 left. The vascular function figure of Ball B-spline reconstruction display of the client is shown in Figure 13 right. Mobile customer interface is shown in Figure 14. Figure 14 left is the maximum intensity projection (MIP) of remote observation of slice data structure at mobile phone client, which simulates the vascular DSA angiography image. Figure 14 right is the display of reconstruction detection results of cerebral blood vessels on the mobile phone.

**Figure 15:**
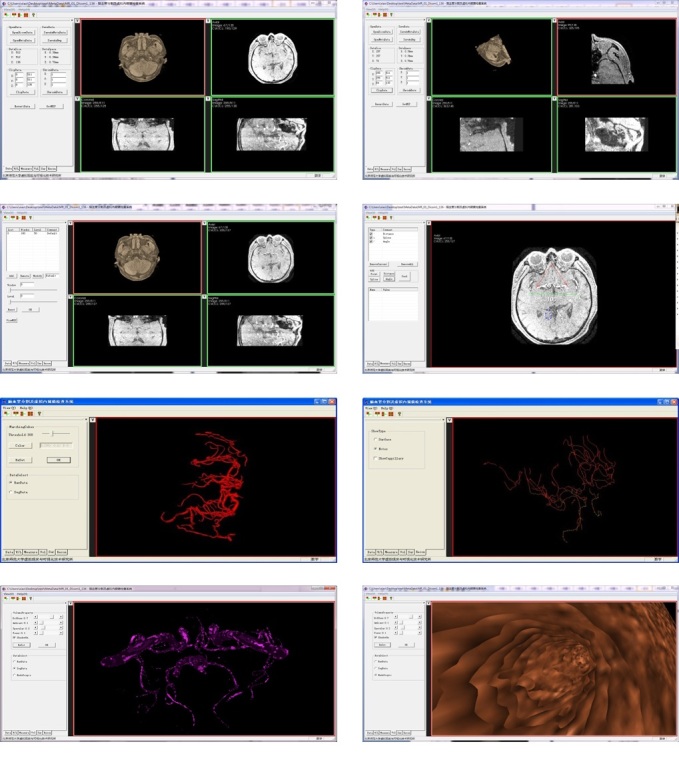
The function introduction of the platform. Descriptions for each row are from left to right. Row 1: an imported image of brain data; the cropping result of the brain image data. Row 2: window and width adjustment of the data with clear view effect; 2D measurement of the brain image. Row 3: statistical segmentation result of the cerebral vessels; reconstruction result of Balls B-spline method. Row 4: volume rendering result; cerebrovascular virtual endoscopic result.

The medical services platform of cerebrovascular diseases at the server side is used for vessel segmentation and reconstruction of the CT and MRI brain images, which mainly uses segmentation algorithm based on statistical methods to finish segmentation and uses Ball B-spline surfaces on vascular reconstruction. At last, the volume rendering and surface rendering are used to display the vessels after segmentation and reconstruction or the vessels enhanced after adding the capillary vessels. In addition, our platform can also achieve the transformation, processing, and organizational measurement of the DICOM, Raw, and BMP sequence cerebrovascular data. The system can handle many common data structure, and has powerful user interaction and friendly interface which is easy to operate. Figure 15 row 1 left is an imported image of brain data. Figure 15 row 1 right is the cropping result of the brain image data. Figure 15 row 2 left is the window and width adjustment of the data with clear view effect. And Figure 15 row 2 right is a 2D measurement of the brain image. Figure 15 row 3 left is the statistical segmentation result of the cerebral vessels. Figure 15 row 3 right is the reconstruction result of Balls B-spline method. Figure 15 row 4 left is the volume rendering result and Figure 15 row 4 right is the cerebrovascular virtual endoscopic result.

## Discussion

This paper introduces the basic framework and functions of cerebrovascular eHealth electronic medical platform. The platform can carry out the remote diagnosis and test other organs with complex geometric structure, and provide application examples for remote eHealth detection. Users can realize the automatic acquisition, management, detection, and diagnosis of remote information to realize medical information at home, thus it reduces the health care costs and improves the health care at the level of encephalopathy. Our system provides the basic medical image processing functions for browsing, window and width adjustment, cropping, and maximum projection map. Here, we have also proposed some innovative technology in automatic segmentation of cerebral vessels, Ball B-spline reconstruction, and ideographic enhanced volume rendering. Manual and automated search of brain lesions are also achieved through the virtual endoscopic and automatic detection. The system provides network client and mobile client platforms, which can realize any health function at any time. However, we only constructed a basic framework and achieved the basic functions, so we hope to further construct the registration algorithms and realize the automatic monitoring of encephalopathy to develop the function of our platform. The platform is still at the online testing phase with a small sample population, it needs to increase data management and parallel computing ability at the testing stage with a larger population sample. We need to strengthen the application of our system in hospitals and medical schools to improve the stability of the platform in the experiments with a large population.

## Abbreviations

2D: 2-dimensional
3D: 3-dimensional
3DRA: 3-dimensional rotational angiography
3NET: telecommunication network, television network and computer network
AJAX: Asynchronous JavaScript and XML
BBSC: Ball B-Spline Curve
C/S: Client/Server
CT: computed tomography
CTA: computed tomography angiography
CUDA: compute unified device architecture
DICOM: digital imaging and communications in medicine
DWM: directional weighted median
EM: Estimation Maximization
G-MRF: Gaussian-Markov random field model
GPU: graphics processing unit
IOT: Internet of things
JS: JavaScript
MIP: maximum intensity projection
MRA: magnetic resonance angiography
MRF: Markov random field
MRI: magnetic resonance imaging
OOP: object oriented programming
PACS: picture archiving and communication system
RDP: remote desktop protocol
RFID: radio frequency identification devices
ROI: region of interest
SEM: Stochastic Estimation Maximization
SQL: structured query language
TOF: time of flight
VNC: virtual network computing
VOI: volume of interest
VTK: visualization toolkit
